# Forgotten Deficiency: A Case Series Highlighting Atypical Presentations of Scurvy in the 21st Century

**DOI:** 10.1155/carm/2118907

**Published:** 2025-06-23

**Authors:** Mohammed Ayyad, Lilian Tran, Safia Ansari, Dennis Levy, Daniel Matassa

**Affiliations:** ^1^Department of Medicine, Rutgers New Jersey Medical School, Newark, New Jersey, USA; ^2^Department of Oral and Maxillofacial Surgery, Rutgers New Jersey Medical School, Newark, New Jersey, USA

**Keywords:** ascorbic acid, inflammation, malnutrition, pulmonary hypertension, scurvy, vitamin C

## Abstract

Scurvy, caused by vitamin C deficiency, is increasingly recognized in contemporary clinical practice, particularly among vulnerable populations. Despite its historical association with maritime exploration, scurvy persists in patients with malnutrition, alcoholism, psychiatric disorders, and limited access to fresh produce. This report presents two cases of scurvy diagnosed in a low socioeconomic urban setting, emphasizing the diverse and atypical manifestations of this condition. The first case involves a 76-year-old female presenting with altered mental status and pulmonary hypertension, ultimately attributed to severe vitamin C deficiency. Echocardiography revealed a pulmonary artery pressure of 36 mmHg and severe tricuspid regurgitation. A thorough evaluation, combined with evidence of malnourishment and neuropsychiatric symptoms, led to the diagnosis of scurvy, confirmed by undetectable serum vitamin C levels. High-dose intravenous vitamin C supplementation resulted in marked clinical improvement and resolution of cardiopulmonary abnormalities. The second case describes a 68-year-old male with alcohol use disorder presenting with syncope, anemia, and systemic inflammation. Laboratory tests revealed leukocytosis, acute kidney injury, elevated ferritin, and undetectable iron-binding capacity. A nutritional workup identified severe vitamin C deficiency (0.2 mg/dL). Oral vitamin C supplementation improved inflammatory markers, anemia, and general well-being. These cases highlight the importance of considering scurvy in patients with unexplained systemic symptoms and malnutrition. Early diagnosis and prompt treatment with vitamin C supplementation can lead to full recovery and prevent severe complications. Clinicians should maintain a high index of suspicion for scurvy, especially in at-risk populations with atypical clinical presentations.

## 1. Introduction

Vitamin C deficiency, also known as scurvy, plagued human populations for centuries. The first written description of a scurvy case dates back to 1500 BC by the Egyptians. During the Age of Exploration, scurvy became a significant source of sickness and mortality throughout most of Europe [1]. Experienced sailors, such as Captain James Cook—highly regarded for preventing scurvy in his sailors—spent months at sea eating a diet rich in vegetables and citrus fruits [2]. As awareness of the importance of fresh fruits and vegetables in the diet increased, the prevalence of scurvy began to decline in the 18th century. With the modernization of nutritional supply and food supplements, scurvy's prevalence continued to significantly decline, and it is now considered a rare disease of antiquity [3].

In the era of modern medicine, the prevalence of scurvy varies worldwide and between studies. In the United States, it has been estimated that 7 ± 0.9% of the population suffers from scurvy, while in North India, the rate is 73.9%. Risk factors for scurvy include biological, psychological, and environmental factors such as chronic alcohol use, tobacco use, poor dietary habits, limited access to or inability to afford fresh fruits and vegetables, low socio-economic status, elderly individuals on a “tea-and-toast” diet, eating disorders, psychiatric illnesses, obesity, and abdominal surgeries [4]. Although many processed foods are fortified with ascorbic acid, these sources are often insufficient to maintain adequate serum levels in individuals with limited intake of fresh produce. Moreover, vitamin C is water-soluble and heat-labile, and its content can be degraded during food processing and storage [5]. Patients in industrialized settings may still develop deficiency due to poor food quality, chronic alcohol use, cognitive or psychiatric disorders, or functional impairments that prevent them from shopping for or preparing nutritionally complete meals [6].

Typical clinical manifestations of vitamin C deficiency can include hematologic, dermatologic, or immunologic dysfunctions. Symptoms can begin to appear after 4–12 weeks of inadequate dietary intake, presenting as non-specific symptoms such as fatigue, anorexia, and irritability. These symptoms can develop when serum concentrations fall below 20 μmol/L, with substantial deficiency more likely at levels below 11.4 μmol/L [4].

Importantly, atypical clinical manifestations, including pulmonary, cardiac, and gastrointestinal symptoms, can often obscure the diagnosis of scurvy. There have been a handful of case reports linking scurvy to pulmonary hypertension (PH) and right-sided heart failure [7]. Theories describing this mechanism involve vitamin C's role in nitric oxide (NO) synthesis. Low endothelial NO levels lead to loss of vasodilatory effects in pulmonary circulation [8]. Additionally, vitamin C's role in iron absorption is another key factor in this mechanism. Hypoxia-inducible family transcription factors (HIF TF) mediate cellular responses to low oxygen conditions and are dependent on both vitamin C and iron. Deficiencies in either can disrupt the regulation of these factors, leading to widespread pulmonary vasoconstriction [7, 9]. Another atypical manifestation includes gastrointestinal bleeding, given vitamin C's role in wound healing and collagen synthesis in vasculature. Due to the high vascularity and large surface area, the GI tract is commonly investigated in patients presenting with anemia, and there have been infrequent cases reporting scurvy presenting as overt gastrointestinal bleeding [2, 10]. These were often seen during endoscopic procedures, showing intramucosal hemorrhages.

Overall, given the broad clinical manifestations of scurvy and the rarity of the disease today, it can often go unnoticed. This is further complicated by the lack of emphasis on dietary history in clinical training, especially when managing vulnerable populations, often resulting in missed cases of vitamin C deficiency, particularly when symptoms overlap with other etiologies such as the cases presented. Here, we present two rare cases of scurvy diagnosed in a low socio-economic urban area within the US, each with distinct presentations and clinical manifestations. In one case, the patient is a 76-year-old female presenting with altered mental status (AMS) and PH of unknown cause. The second case is a 68-year-old male with typical risk factors for scurvy, presenting with syncope thought to be secondary to acute anemia. Although both patients were considered part of a high-risk population, there was a low index of suspicion for scurvy due to their nonspecific presentations and the rarity of the disease. However, thorough history-taking and consideration of broad differentials led to a successful diagnosis of scurvy, confirmed by low or undetectable serum vitamin C levels, and appropriate treatment enabled full clinical recovery.

## 2. Case Presentation

### 2.1. Case 1

A 76-year-old female presented to the hospital with AMS, brought by EMS after being found confused at Newark Airport. The patient had recently flown in from India, where she stated she had been for the past six months on a “spiritual trip.” She was unable to provide a coherent history, displaying paranoia, resistance to questioning, and vague statements such as “the boys put me on the plane involuntarily.” Despite this, she was alert, awake, and oriented to person, place, and time. She denied any significant past medical history and was noted to be homeless and malnourished. Her family and social situation were unclear, and she had no known medications on record. EMS reported that she was disoriented and uncooperative upon arrival.

**Figure 1:**
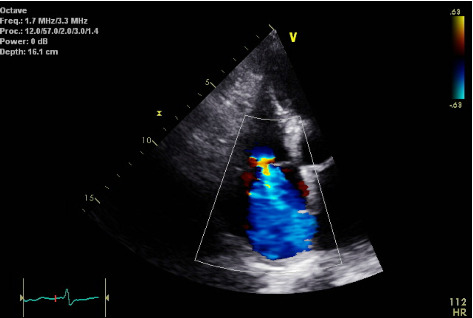
Color Doppler echocardiographic image showing turbulent regurgitant jet across the tricuspid valve with a regurgitant velocity of 264 cm/s and an assumed right atrial pressure of 8 mmHg. These findings are suggestive of severe tricuspid regurgitation.

In the emergency department, her vital signs were notable for a blood pressure of 151/93 mmHg, a heart rate of 157 beats per minute, and a respiratory rate of 18 breaths per minute. She was afebrile. An initial electrocardiogram (EKG) revealed atrial fibrillation with rapid ventricular response. Labs were significant for mild hyponatremia, hypokalemia, and mild anemia, however, otherwise unremarkable including a normal TSH. Urinalysis was positive for a urinary tract infection, while the urine toxicology screen was noncontributory. In addition, a D-dimer was elevated at 1524 ng/mL, and brain natriuretic peptide (BNP) was elevated. On imaging, CT scan of the head showed an incidental meningioma without acute intracranial pathology. Further imaging with a CT pulmonary angiogram was negative for pulmonary embolism but showed cardiomegaly and small pericardial recess fluid. A subsequent transthoracic echocardiogram revealed PH with a pulmonary artery pressure of 36 mmHg, severe tricuspid regurgitation (TR) with a regurgitant velocity of 264 cm/s, and right atrial enlargement with chamber pressure of 8, and normal LVEF and left-sided pressures (Figure 1). On skin examination, the patient was noted to have bilateral lower extremity petechial rash and signs of malnourishment, including alopecia, dehydration, and muscle atrophy.

Given her AMS and urinary tract infection, blood cultures were drawn, and IV ceftriaxone was initiated. She was also started on rate control therapy with metoprolol. The primary team decided to further investigate potential causes of PH based on her echocardiogram findings. Infectious, vascular, and connective tissue disorder workups were all negative. Considering her recent travel to India, neuropsychiatric symptoms, and objective evidence of malnourishment, scurvy-induced PH was suspected. Subsequently, nutritional evaluation was conducted, and vitamin C level was ordered, which was found to be undetectable at < 0.1 mg/dL. She was started on high-dose intravenous vitamin C supplementation with gradual improvement of her clinical status and shortness of breath.

On hospital day eight, the patient's clinical course continued to improve, and she was deemed stable for discharge, with plans for echocardiographic follow-up at an outpatient cardiology clinic. However, the patient was unfortunately lost to subsequent follow up. This represents a notable limitation, as no follow-up echocardiographic data were available to confirm resolution of PH. Although her clinical symptoms improved, this does not confirm reversal of the underlying hemodynamic abnormality as would be expected in scurvy-induced PAH.

### 2.2. Case 2

A 68-year-old male with a history significant for alcohol use disorder and coronary artery disease presented to the emergency department after a syncopal episode while grocery shopping. He reported drinking one pint of gin a day for the past 30 years and has a 10-pack-year history of smoking. His last alcohol intake was early the previous morning. He reported chronic fatigue, weakness, and pain in his toes and feet. Of note, he described poor access to food and mentioned his inability to cook for himself.

On presentation in the emergency room, vital signs were stable. On physical exam, the patient appeared cachectic with dry mucous membranes and poor dentition. The abdomen was diffusely tender without guarding or rebound tenderness. His feet were dry without lesions or ulcers but exhibited numbness, tenderness, and sensitivity in all toes. Initial labs (Table 1) were significant for significant hypokalemia, anion gap metabolic acidosis, hypokalemia, hypophosphatemia, low vitamin D and elevated ferritin. The total iron-binding capacity was undetectable, and inflammatory markers were elevated. CBC showed leukocytosis, acute on chronic anemia, and thrombocytopenia. Creatinine was elevated to 3.4 from a baseline of 1.0.

An extensive workup including blood cultures, urinalysis, and imaging studies were unremarkable. Hepatitis panel, rapid plasma regain, antineutrophil cytoplasmic antibody, rheumatoid factor, myeloperoxidase antibodies, cyclic citrullinated peptide antibodies, anti-glomerular basement membrane antibodies, lactate, urine toxicology screen, and haptoglobin were all within normal limits. Additional laboratory evaluation included a creatine kinase level of 298 U/L (reference: 0–200 U/L), and a urinalysis, which was negative for infection or myoglobinuria. Liver function tests revealed a mildly elevated AST of 43 U/L and total bilirubin of 1.1 mg/dL.

Given the patient's history of malnutrition, empiric supplementation of vitamin C, D, and folate was started, while a nutritional workup was done to evaluate for deficiency. Subsequently, vitamin C levels were markedly low at 0.2 despite initial empiric therapy. The patient was subsequently managed with continued vitamin supplementation, and IV hydration to manage acute kidney injury. Ultimately, the patient's leukocytosis resolved, and the inflammatory markers continued to downtrend without further intervention. He was subsequently discharged with continued supplementation of vitamin C in addition to other vitamins.

Notably, the patient did not exhibit classic mucocutaneous features of scurvy, such as petechiae, perifollicular hemorrhages, or gingival bleeding. The absence of these hallmark findings weakens diagnostic specificity and suggests that vitamin C deficiency in this case may have acted as one of several contributors to the inflammatory and constitutional symptoms rather than the sole etiology.

## 3. Discussion

Scurvy, a disease long thought to be relegated to the annals of maritime history, is re-emerging in modern clinical practice, particularly in vulnerable populations. This resurgence is often seen among individuals with malnutrition, alcoholism, psychiatric disorders, or limited access to fresh produce. Our case series illustrate the diverse and atypical manifestations of scurvy, emphasizing the importance of recognizing this condition in patients who may not present with the classic symptoms traditionally associated with historical vitamin C deficiency.

### 3.1. Pulmonary and Cardiovascular Manifestations

The first case of a 76-year-old female with PH and severe TR highlights the lesser-known cardiovascular sequelae of scurvy. Vitamin C plays a pivotal role in maintaining vascular integrity and modulating endothelial function through its involvement in NO synthesis [8]. NO is a potent vasodilator essential for maintaining pulmonary vascular tone [11]. In vitamin C deficiency, reduced NO availability leads to vasoconstriction, contributing to elevated pulmonary artery pressures and, subsequently, right-sided heart dysfunction [12, 13].

PH secondary to scurvy is increasingly recognized in the literature. In a case series by Gayen et al. patients with severe vitamin C deficiency exhibited pulmonary artery pressures as high as 76 mmHg. After ascorbic acid supplementation, pulmonary pressures decreased significantly, highlighting the reversibility of scurvy-induced PH [12]. This patient population, characterized by poor nutritional intake and elevated risk factors, often demonstrates right heart strain and TR, as seen in our case. Echocardiographic follow-up is crucial to monitor the resolution of PH postsupplementation.

A study by Gaietto et al. further demonstrated the reversibility of scurvy-induced PH, were a four-year-old child with undetectable vitamin C levels and severe malnutrition exhibited a pulmonary artery pressure of 52 mmHg, which normalized following parenteral nutrition and vitamin C supplementation [13]. This study highlights that scurvy-induced cardiopulmonary complications are not age-restricted and can present in diverse clinical contexts. Furthermore, the rapid and drastic response of vitamin C supplementation highlights the importance of having it in the differential of PAH, given that supplementation can lead to complete recovery. In our case, the patient was scheduled for a repeat echocardiogram; however, they were lost to further follow up. This represents a significant limitation of the case. Without follow-up echocardiographic data, it is not possible to objectively confirm resolution of PH. While the patient's clinical improvement was notable, hemodynamic reversal remains speculative and cannot be solely attributed to vitamin C supplementation.

Additionally, it is important to consider potential confounders in interpreting the patient's PH. Atrial fibrillation with rapid ventricular response and systemic infection may transiently elevate right-sided pressures through increased cardiac output or inflammatory vasoconstriction. That said, scurvy remains a compelling etiology in this case due to the absence of structural heart disease, negative autoimmune and infectious workup, and the presence of profound biochemical vitamin C deficiency with classic dermatologic and nutritional signs and notable temporal imporovement with suppelementation of high doses of vitamin C. Moreover, there is growing literature linking vitamin C deficiency to PH through impaired NO synthesis and dysregulation of hypoxia-inducible pathways, which are both vitamin C–dependent. Differentiating between transient hemodynamic effects and sustained pulmonary vascular pathology is essential, and in this patient, the follow up echocardiogram would have been a great tool to differentiate between both etiologies.

### 3.2. Inflammatory and Hematologic Abnormalities

The second case involved a 68-year-old male with anemia, elevated inflammatory markers, and leukocytosis of unclear etiology, which have resolved after vitamin C supplementation. According to the literature, vitamin C, a potent antioxidant, appears to exert anti-inflammatory effects by scavenging free radicals and inhibiting monocyte apoptosis, which leads to reduced systemic inflammation [14]. Ding et al. demonstrated a significant inverse relationship between high-sensitivity C-reactive protein (hs-CRP) and ascorbic acid levels in a cross-sectional analysis of 5380 participants [15]. This is further corroborated by two other studies demonstrating an inverse relationship between vitamin C and CRP levels [16, 17]. A clinical trial conducted by Block et al. found that vitamin C supplementation (1000 mg/day) significantly reduced CRP levels by 25.3% in individuals with elevated CRP (≥ 1.0 mg/L) [16]. This relationship demonstrates the potential utility and crucial role of vitamin C in mitigating chronic inflammation, suggesting that patients presenting with unexplained high inflammatory states may benefit from nutritional evaluation and supplementation if deficient.

In our case, despite extensive infectious and autoimmune workups, no etiology for the patient's persistent inflammation was identified, prompting a nutritional assessment that revealed vitamin C deficiency. It is noteworthy to mention that the patient's regular alcohol consumption could also have contributed to elevated inflammatory markers. However, the observed temporal association between the decline in inflammatory markers after initiating vitamin C supplementation suggests a potential role of vitamin C deficiency in our patient's heightened inflammatory state. That said, the degree of elevation of inflammatory markers was disproportionately high for scurvy alone and raises the possibility of coexisting processes. Mild elevations in creatine kinase (298 U/L) and AST (43 U/L) suggest a component of low-grade muscle injury or alcohol-related hepatocellular stress, though not diagnostic of rhabdomyolysis or hepatitis. Additionally, while urinalysis was negative and liver function abnormalities were minor; these values should prompt clinicians to consider alternative or concurrent pathology in similarly complex patients.

Furthermore, the absence of classic mucocutaneous findings—such as petechiae, purpura, or gingival bleeding—in this patient weakens diagnostic certainty. While vitamin C deficiency was confirmed biochemically and supplementation led to clinical improvement, the lack of hallmark cutaneous signs highlights that scurvy in modern presentations may often exist as part of a broader picture of malnutrition, systemic stress, and chronic disease.

## 4. Conclusion

This case series highlights the re-emergence of scurvy in contemporary clinical practice and its atypical, often underrecognized manifestations, including PH and systemic inflammation. In both cases, vitamin C deficiency was biochemically confirmed and temporally associated with clinical improvement. However, the presence of concurrent conditions—such as atrial fibrillation, infection, alcohol use, and malnutrition—necessitates a cautious interpretation of causality.

That said, these cases highlight that scurvy may act as a contributory rather than exclusive driver of systemic pathology, particularly in vulnerable populations and should be evaluated and treated if deficient. In the first case, severe vitamin C deficiency was one of few plausible etiologies for PH after an unrevealing workup. In the second, it may have exacerbated a broader inflammatory and nutritional derangement.

Clinicians should maintain a high index of suspicion for vitamin C deficiency in patients presenting with unexplained cardiopulmonary symptoms, inflammation, or anemia—particularly in the context of poor nutrition, alcohol use disorder, or functional food insecurity. Even in the absence of classic cutaneous signs, nutritional assessment and empiric repletion can be both diagnostic and therapeutic. Given its low cost, wide safety margin, and potential for reversibility of serious complications, vitamin C supplementation is a high-yield intervention when deficiency is suspected.

Ultimately, these cases reinforce the importance of integrating dietary history and nutritional risk assessment into routine clinical evaluation—especially in underserved populations—where forgotten deficiencies may still present with full clinical force.

## Figures and Tables

**Table 1:** Laboratory values on admission and one week postdischarge in a patient with scurvy.

Lab	On admission (9/22/24–9/23/24)	One week after discharge (10/5/24)
Hemoglobin	5.2 (L)	7.6 (L) g/dL
Platelet count	88 (L)	282 × 10^3^/µL
White blood cells	12.2 (H)	9.1 × 10^3^/µL
Potassium	1.8 (L)	4.2 mmol/L
Calcium	5.2 (L)	8.0 (L) mg/dL
CO_2_ (bicarbonate)	19 (L)	23 mmol/L
Phosphorus	2.1 (L)	3.1 mg/dL
Vitamin D (25-OH)	4.9 (L)	6 ng/dL
Parathyroid hormone (PTH)	505.1 (H)	Not drawn
Folate	4.2 (L)	Not drawn
Ferritin	2493 (H)	765 ng/mL
TIBC (total iron binding capacity)	< 157 (L)	227 μg/dL
C-reactive protein	114 (H)	44 (H) mg/L
Erythrocyte sedimentation rate	92 (H)	Not drawn
Creatinine	3.1 (H)	1.0 mg/dL

## Data Availability

The data that support the findings of this study are available from the corresponding author upon reasonable request.
